# A Supplemental Women’s Health Questionnaire for Women Veterans With Military Environmental Exposures: Project Development and Implementation

**DOI:** 10.2196/73223

**Published:** 2025-07-24

**Authors:** Leah N Eizadi, Mehret T Assefa, Jordan M Nechvatal, G Marina Veltkamp, Abou Ibrahim-Biangoro, Maheen M Adamson, Jennifer S Jennings

**Affiliations:** 1 California War Related Illness and Injury Study Center, VA Palo Alto Health Care System, United States Department of Veterans Affairs, Palo Alto, CA, United States; 2 Women's Operational Military Exposure Network Center of Excellence, VA Palo Alto Health Care System, United States Department of Veterans Affairs, 3801 Miranda Ave, Palo Alto, CA, 94304, United States, 1 888 482 4376; 3 Department of Neurosurgery, School of Medicine, Stanford University, Stanford, United States

**Keywords:** women, veterans, reproductive health, women’s health, questionnaire, military environmental exposures

## Abstract

**Background:**

The number of women in the armed forces has steadily increased across all branches, even as the overall size of the military remains stable. The population of women veterans is also expanding. The existing literature has extensively reported the impact of military environmental exposures (MEEs) on adverse physical and mental health outcomes in service members and veterans; however, most of these studies focus on the experiences of men. In response to the growing need to address women-specific health care concerns, particularly for women with MEEs, the Women’s Operational Military Exposure Network Center of Excellence (WOMEN CoE) developed and implemented the Women’s Health Addendum (WHA).

**Objective:**

The primary objective of this project is to (1) describe the development and implementation of a comprehensive health questionnaire for women veterans, (2) systematically describe and characterize the health conditions of women seeking care for MEE-related health concerns, and (3) use findings to inform clinic policies and develop targeted programs.

**Methods:**

The WHA was introduced to assess the prevalence of health conditions that are female-specific, or disproportionately impact women; examine the relationship between these health conditions and MEEs; and use findings to improve care. The WHA was developed through an iterative process, incorporating literature review, veteran and clinician feedback, and clinical expertise. It consists of 81 questions across 7 categories related to health conditions across the lifespan and was implemented in 2 phases. Phase 1 was administered to women at the California War Related Illness and Injury Study Center (WRIISC), and phase 2 included women at the New Jersey and Washington, DC, WRIISC sites. Descriptive findings are presented here.

**Results:**

A total of 63 women participated in the program evaluation from October 2022 to April 2024. In phase 1, 39% (29/75) of the women who were invited agreed to participate. In phase 2, 34 (10%) of the 325 invited veterans responded. Several women’s health conditions were reported, with approximately 97% (61/63) of women reporting at least one health condition and 87% (55/63) reporting 3 or more. Among respondents, the most prevalent conditions included sexual dysfunction (23/33, 70%), urinary incontinence (33/56, 59%), pelvic floor dysfunction (33/63, 52%), and pregnancy loss (20/45, 44%). Overall, more than 40% (3/7) of the most frequent conditions were related to urinary health and pelvic floor dysfunction.

**Conclusions:**

Findings highlight the need for services related to women’s health, especially for this cohort with MEE concerns seen at a tertiary care center. Initial findings emphasize concerns that women have about fertility and MEE experienced during deployments. Next steps include administering the WHA to women at sister WRIISC sites in real time and establishing a wider distribution network for the WHA. Future efforts to further evaluate the relationship between MEE and women’s health concerns are underway.

## Introduction

### Women Veterans

With over 2 million US women veterans, women are the fastest-growing segment of the veteran population and are projected to be 18% of the population by 2040 [1]. According to the Department of Veterans Affairs (VA), women are also increasingly using VA services [1]. At various fiscal year time points, the number of women using VA services nearly doubled or tripled, while the number of men grew substantially slower over the same period [2,3]. This increase represents a more than 300% growth among women using VA primary care [3], yet despite these improvements, women are still a minoritized group with less than 30% of all women veterans enrolled in VA services [2].

Within the VA, women are more likely to have higher outpatient utilization, be younger, and represent a more racially or ethnically diverse group compared to men [2]. For women VA patients, the most prevalent health conditions are endocrine/metabolic/nutritional, musculoskeletal, mental health/substance use disorders, reproductive health, neurologic, sense organ, cardiovascular, and gastrointestinal [3]. For reproductive and sexual health conditions, the most frequent diagnoses include urinary disorders, breast conditions and reproductive organ disorders, menstrual and menopausal disorders, contraceptive care management, and sexually transmitted diseases [4]. As VA projects the resources needed for the future care of the expanding women veteran population, clinical and educational efforts must consider the health problems faced by women.

### Military Service and Military Environmental Exposures

Although women have served in the US armed forces for over 200 years, it was not until 1948 that women were made permanent members of all branches of the armed forces, and not until 2013 that women were allowed to serve in combat roles [5]. As the role of women in the military has changed over time, so has their exposure to military environmental exposures (MEEs). Examples of MEEs that women may have been exposed to during military service include air pollutants, chemicals, radiation, occupational hazards, and chemical warfare agents [6]. For women, exposures during military service have been increasing since the 1990s due to changes in policy, length of conflicts, and expanded roles in the military [5,7].

A growing body of literature indicates that an association between MEEs and adverse health problems exists. For example, research on MEEs, physical health, and mental health conditions includes chronic multisymptom illness or Gulf War illness [8,9], impairments in activities of daily living and physical functioning [10], peripheral neuropathy and widespread pain [11], cognitive functioning [12,13], depression [14], anxiety and posttraumatic stress disorder symptoms [14,15], and quality of life [14]. However, most of the MEE literature is based on men and often focused on Vietnam War–era or Gulf War I–era service members and veterans [16]. Although studies have highlighted differences in military service and deployment effects between men and women, more studies are needed to better understand the postdeployment health of women veterans [17-21]. As women transition from service members to veterans, their risk for health concerns that are female-specific or disproportionately impact women may increase, yet few studies have been conducted with women veterans, and even fewer have focused on their reproductive health [22].

### Current Status and Gaps

Most of the research conducted among women veterans, or examining sex differences among veterans from the Gulf War and post-9/11 eras, has been observational studies focused on mental health, such as posttraumatic stress disorder, military sexual trauma, and substance use disorders [19,23]. While the number of studies on women veterans is growing, several reviews have reported that gaps in the extant literature on women’s health remain and have highlighted the lack of sex-specific results from studies on all veterans [18,23-25]. Many of the reproductive health studies among women veterans used clinical records or qualitative interviews (vs self-reported questionnaires) or examined specific age-related reproductive health issues such as contraception or pregnancy (vs multiple conditions across the lifespan) [22,23]. Topics such as infertility and menopause have been identified as key knowledge gaps in the reproductive health literature, and the need to assess reproductive health outcomes using standardized measures has been highlighted [22]. To our knowledge, there are no standardized women’s health questionnaires for women veterans.

### WRIISC and WOMEN CoE

**Figure 1.**
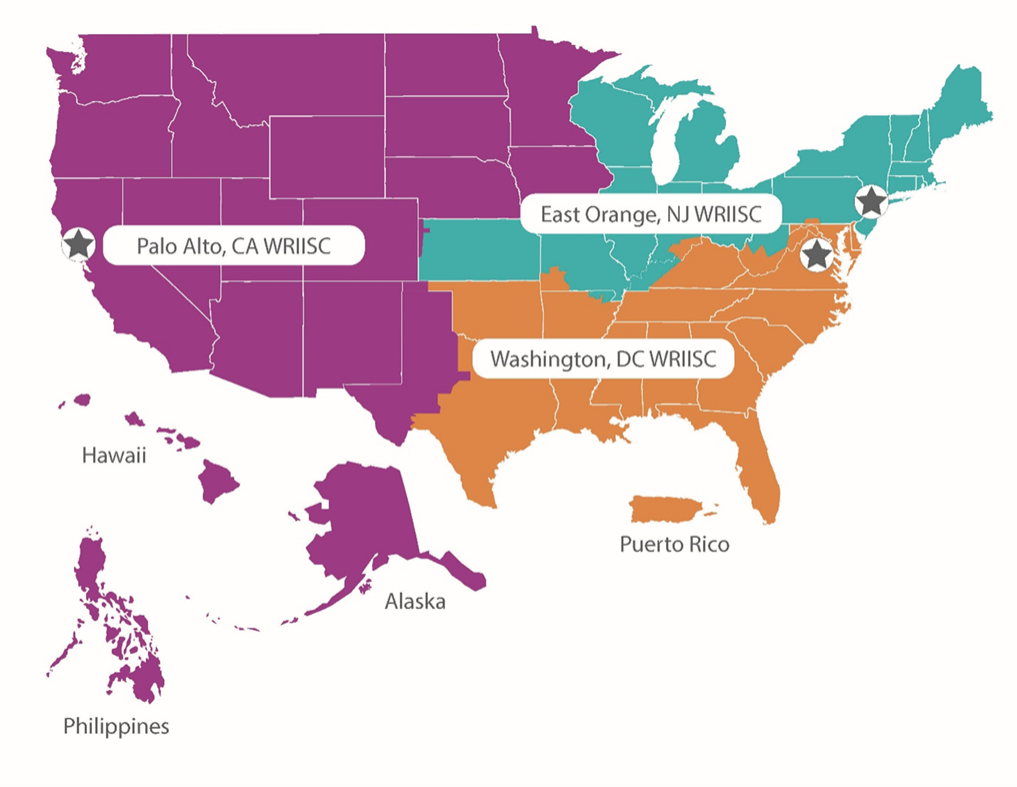
WRIISC | WOMEN CoE clinical service areas. Clinical services for the Women’s Operational Military Exposure Network Center of Excellence (WOMEN CoE) are provided by the War Related Illness and Injury Study Center (WRIISC).

Established in 2001, the War Related Illness and Injury Study Center (WRIISC) is a national tertiary care center at VA using a multidisciplinary translational approach to the care of veterans with MEE concerns [6,26,27]. There are 3 sites across the United States (California [CA], New Jersey [NJ], and Washington, DC [DC]) that provide postdeployment health services for the veterans in their catchment areas, including clinical care, education, and research (see Figure 1) [6,26].

**Figure 2.**
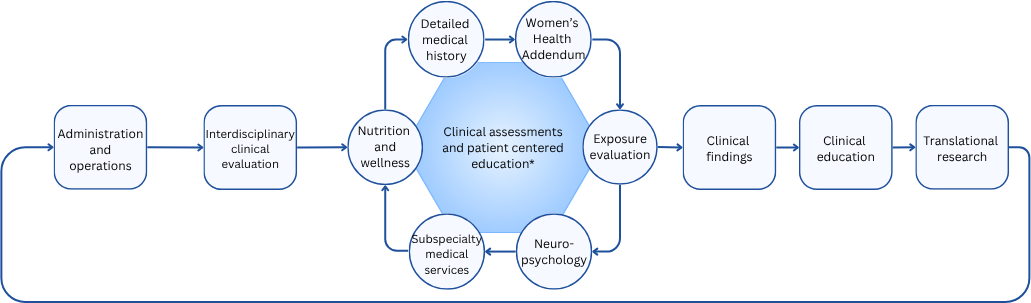
WOMEN CoE clinical process flowchart. This clinical process flowchart shows the different components that make up the Women’s Operational Military Exposure Network Center of Excellence (WOMEN CoE) clinical services. Except for the Women’s Health Addendum (WHA), clinical services are provided by the War Related Illness and Injury Study Center (WRIISC).

In 2022, VA established the Women’s Operational Military Exposure Network (WOMEN) to address the growing health concerns of women veterans with MEEs, and in 2024, it was designated a Center of Excellence (CoE) [28,29]. In alignment with WRIISC, WOMEN CoE provides a multipronged approach to the care of women veterans with the goal of evaluating the health of women with MEE concerns, investigating the impact of MEEs, and offering resources to providers, patients, and key stakeholders [28]. In this report, we describe the activities of the clinical care pillar, which has been described elsewhere (see Figure 2) [28]. Briefly, WOMEN CoE clinical services are provided by WRIISC clinicians during week-long virtual appointments. The Women’s Health Addendum (WHA), a clinical tool designed to assess health conditions that are female specific or disproportionately impact women, was developed and implemented by WOMEN CoE.

### Objective

The primary objective of this project is to (1) describe the development and implementation of a comprehensive health questionnaire for women veterans, (2) systematically describe and characterize the health conditions of women seeking care for MEE-related health concerns, and (3) use findings to inform clinic policies and develop targeted programs to address the most common health concerns among women veterans seen at the WRIISC.

## Methods

### Women’s Health Addendum

#### Rationale

The WRIISC intake packet was created in 2012 and contains questions on demographics, health concerns, military and deployment information, MEE history, and standardized questionnaires to assess cognitive difficulties, mental health, and physical functioning. The primary purpose of this tool is to aid WRIISC clinicians in their assessment of veterans assigned to a comprehensive, 1-week evaluation. When this questionnaire was originally designed, the target population was primarily men. Since then, the number of women accessing VA services and seeking treatment at the WRIISC has been steadily increasing. While the WRIISC intake packet addressed a wide range of topics, gaps in topics related to women’s health existed. Additionally, women veterans across different ages and deployment eras voiced their concerns about MEEs and how they impact their reproductive and sexual health. To effectively address these concerns, the WHA was introduced.

#### Development of WHA

Development of the WHA began in the summer of 2022. Led by the nurse practitioner, a small group at WOMEN CoE began assessing what was missing from the WRIISC intake packet regarding women’s health. Following the initial assessment, an extensive literature review was conducted to examine what questionnaires on women’s health, deployment, and MEEs already existed. The literature review included studies of women veterans from the Gulf War and post-9/11 eras as nearly 70% (51/76) of the women seen at WRIISC have served during one of these eras.

Studies with a reproductive health focus were reviewed to assess the scope of the problem and identify the health issues most frequently reported among women veterans [22,30]. Studies that used questionnaires to assess self-reported women’s health were examined to either (1) identify a standardized questionnaire or (2) generate a list of topics covered or questions asked to be included in the WHA [17,31]. In studies that used questionnaires to assess women veterans’ reproductive health, standardized assessments were not used.

In addition to the literature review, the preliminary version of the WHA included conditions added by the clinical team that were deemed clinically relevant (eg, miscarriages and reproductive cancers) or were frequently discussed during comprehensive visits (eg, incontinence and sexual function).

During pilot testing, the preliminary version of the WHA was administered via video appointments to CA WRIISC women who were attending the week-long telehealth comprehensive appointments. The addendum was administered by the nurse practitioner or the clinical director, and consent was obtained prior to administration. Veteran and clinician feedback was solicited at the end of each appointment. Additional edits were made to the WHA to improve comprehension, ensure comprehensiveness, and maintain a reasonable length.

Based on findings from the literature, feedback from women veterans and clinicians, and clinical expertise, the WHA was developed through an iterative process and designed to work in conjunction with the WRIISC intake packet. The purpose of the WHA is to (1) assess the prevalence of health conditions that are female-specific or disproportionately impact women across the tri-WRIISC, (2) examine the relationship between these health conditions and MEEs, and (3) use findings to inform clinic policies and develop targeted programs to address the most common health concerns among women veterans seen at the WRIISC.

#### Questions in WHA

Currently, the WHA questionnaire consists of 81 adaptive multiple-choice, yes-no, and fill-in-the-blank questions across 7 categories: sexual health, endocrine health, reproductive health, pregnancy, urinary health, mental health, and menopause. The questions are related to health concerns that are female-specific or that disproportionately impact women across the lifespan. For example, there are questions on age-related conditions (eg, infertility or menopause) and questions on health conditions that can occur at any age (eg, urinary health or sexual health). Descriptions of the categories and sample questions for each are available in Table 1.

**Table 1.** Categories and sample questions from the Women’s Health Addendum questionnaire.

Categories	Description	Sample question
Sexual health	Questions related to sexual functioning and pelvic floor dysfunction	Have you experienced pelvic floor dysfunction such as chronic pelvic pain or pain with intercourse?
Endocrine health	Questions related to endocrine complications such as endometriosis and polycystic ovary syndrome (PCOS)?	Have you been diagnosed with PCOS?
Reproductive health	Questions related to infertility and cancers	Have you experienced issues with fertility?
Pregnancy	Questions related to pregnancy and pregnancy-related complications	Did you experience gestational diabetes?
Urinary health	Questions related to urinary health such as urinary incontinence and urinary tract infections (UTIs)	Did you experience UTIs during your deployment?
Mental health	Questions related to mental health during pregnancy and menstruation	Have you experienced prenatal depression?
Menopause	Questions related to menopause and postpregnancy, including menopause diagnosis and treatments, and major surgical procedures such as hysterectomy	Have you used, or are you currently using, hormone replacement therapy?

The WHA is organized by category and employs branching logic to improve questionnaire flow and display relevant questions. Due to the sensitive nature of the topics covered, participants can skip questions that are triggering or elicit an unwanted reaction. As a note, because this is an addendum to the WRIISC intake packet, questions on exposures, military history, and other health conditions are not included.

### Population

Participants in the WOMEN CoE program evaluation project are WRIISC clinical patients who had a referral to one of three WRIISC sites. Specifically, eligible women veterans are those with medically unexplained symptoms lasting for more than 6 months, difficult-to-diagnose postdeployment health concerns, an overseas deployment, and concerns about MEEs [6,28]. Additional requirements include a completed WRIISC intake packet. WOMEN CoE participants are more likely to be older (over 50 years old), enlisted, Army, non-Hispanic White, and post-9/11 veterans. Additionally, as a tertiary care clinic, WRIISC clinical patients have more complex health problems and are more likely to use VA services than the general veteran population.

### Procedures

**Figure 3.**
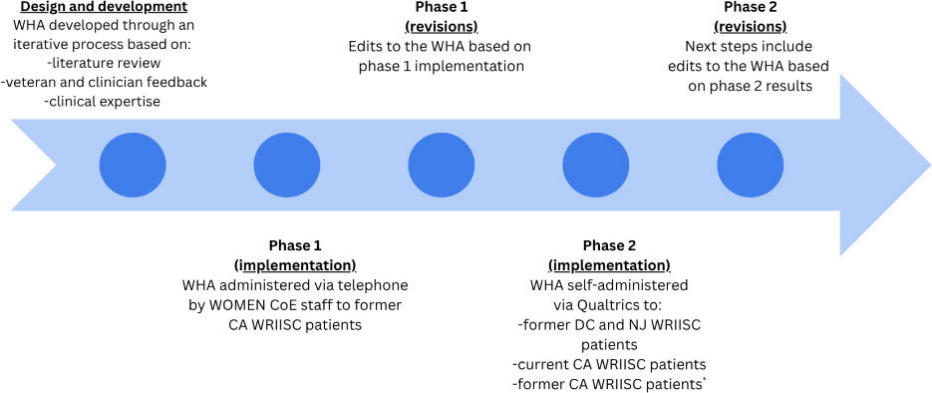
Implementation of the Women’s Health Addendum, 2022-2024. CA: California; DC: Washington, DC; NJ: New Jersey; WHA: Women’s Health Addendum; WOMEN CoE: Women's Operational Military Exposure Network Center of Excellence; WRIISC: War Related Illness and Injury Study Center. *Former CA WRIISC patients who were not successfully reached during phase 1.

The WHA was implemented in 2 phases (see Figure 3). In phase 1, WOMEN CoE staff sent introductory letters to former CA WRIISC patients who had completed the WRIISC intake packet announcing the newly established WOMEN CoE, describing the WHA, and explaining its purpose. The letter was signed by the CA WRIISC clinical director and sent via the veteran’s patient portal. Following the letter, WOMEN CoE staff invited veterans via phone to complete the WHA. For veterans who agreed to participate, the questionnaire was administered by phone with WOMEN CoE staff, and responses were entered into Qualtrics. Due to the sensitive topics covered in the WHA, women were also provided the option to complete the questionnaire on their own via Qualtrics.

At the end of phase 1, a thorough review of the WHA was conducted to assess survey flow, number and type of questions, and ease of comprehension. Additional edits to the survey were incorporated based on patient and staff feedback. Examples of changes made include rearranging questions within categories, adding questions to the reproductive health section to include surrogacy, and language adjustments to improve comprehension. Due to the positive veteran feedback and interest from NJ and DC WRIISC leadership, phase 2 was implemented.

In phase 2, the WHA was administered via Qualtrics to former DC and NJ WRIISC patients who had completed the WRIISC intake packet. This option was also used for former CA WRIISC patients who were not successfully reached by phone, and for current CA WRIISC patients. To improve response rates for NJ and DC, introductory letters were accompanied by messages from the respective clinical directors. Phase 2 is ongoing, and additional modifications will be made at the end based on feedback. The Checklist for Reporting Results of Internet e-Surveys (CHERRIES) [32] is available in Checklist 1.

### Ethical Considerations

The Stanford institutional review board has reviewed and determined that this project does not meet the definition of human subject research as defined in federal regulations 45 CFR 46.102 or 21 CFR 50.3.

### Data Analysis

Findings from the WHA are based on data that were collected as part of a larger program evaluation project to improve care among women veterans seen at the WRIISC. Descriptive statistics are presented for selected results. Data were analyzed using SPSS Statistics Version 29 (IBM Inc).

## Results

From October 2022 to April 2024, a total of 63 women veterans completed the WHA. In phase 1, 75 women who had completed the WRIISC intake packet were invited to participate. Of these, 39% (29/75) responded. In phase 2, 34 (10%) of the 325 invited veterans responded.

Table 2 presents self-reported demographic and military characteristics of the sample. More than half (39/63, 62%) of respondents were from the CA WRIISC catchment area, followed by NJ (14/63, 22%) and DC (10/63, 16%) WRIISC sites. The average age was 51.8 years, with 40% (25/63) of women between the ages of 50 and 59 years. Most identified as non-Hispanic White (45/56, 80%) and reported being married (29/59, 49%). Nearly 70% (51/76) served during the post-9/11 (29/76, 38%) or Gulf War I (22/76, 29%) eras. All women in this sample had at least one deployment (range 1‐5), with an average deployment length of 10.1 months. The majority served in enlisted ranks (48/59, 81%) and on active duty (28/49, 57%) across all branches, but primarily in the Army (29/54, 54%) and Air Force (18/54, 33%). Overall, the length in service was an average of 10.6 years, ranging from 0.3 to 28.1 years.

**Table 2.** Demographic and military characteristics of women veterans with military environmental exposures, 2022-2024.^a^

Characteristic	Women (n=63)
Age (years), mean (SD)	51.8 (9.3)
Age (years), n (%)	
30‐39	9 (14)
40‐49	14 (22)
50‐59	25 (40)
60+	15 (24)
Race or ethnicity,^b^ n (%)	
American Indian or Alaskan Native	3 (5)
Non-Hispanic Black	3 (5)
Hispanic or Latino	5 (9)
Non-Hispanic White	45 (80)
Marital status,^c^ n (%)	
Currently married	29 (49)
Divorced or widowed	13 (22)
Never married	11 (19)
Other^d^	6 (10)
Branch of service,^e^ n (%)	
Air Force	18 (33)
Army	29 (54)
Marines	3 (6)
Navy	4 (7)
Military service component,^f^ n (%)	
Active duty	28 (57)
Reserves or guard	8 (16)
Both	13 (27)
Service era,^b^ n (%)	
Gulf War I	22 (29)
Post-9/11	29 (38)
Other combat deployments^g^	9 (12)
Noncombat deployments	16 (21)
Rank,^c^ n (%)	
Enlisted	48 (81)
Officers	11 (19)
Number of deployments,^h^ mean (SD)	1.4 (1.0)
Deployment length^i^ (months), mean (SD)	10.1 (6)
Service length (years), mean (SD)	10.6 (7.6)
WRIISC^j^ site, n (%)	
California	39 (62)
New Jersey	14 (22)
Washington, DC	10 (16)

a Includes self-identified demographic and military characteristics.

b Missing (n=7).

c Missing (n=4).

d Other marital status includes living with a partner, separated.

e Missing (n=9).

f Missing (n=14).

g Other combat deployments non-Gulf War I and post-9/11 deployments such as Bosnia, with some participants serving across multiple eras.

h Missing (n=12).

i Missing (n=16).

j WRIISC: War Related Illness and Injury Study Center.

Several health conditions that are female specific or more prevalent in women were reported (see Table 3). Approximately 97% (61/63) reported at least one health condition, and 87% (55/63) reported 3 or more. Among those who responded, the most prevalent conditions included sexual dysfunction (23/33, 70%), urinary incontinence (33/56, 59%), pelvic floor dysfunction (33/63, 52%), pregnancy loss (20/45, 44%), urinary tract infections (23/55, 42%), uterine fibroids (25/62, 40%), and hysterectomy (22/62, 35%). Overall, 43% (3/7) of the top frequent concerns were related to urinary health and pelvic floor dysfunction.

**Table 3.** Number of most frequently reported women’s health conditions among women veterans with military environmental exposures, 2022-2024 (n=63).^a^

Health conditions	Women, n (%)
Sexual dysfunction (n=33)	
Yes	23 (70)
No	10 (30)
Urinary incontinence (n=56)	
Yes	33 (59)
No	23 (41)
Pelvic floor dysfunction (n=63)	
Yes	33 (52)
No	30 (48)
Pregnancy loss (n=45)	
Yes	20 (44)
No	25 (56)
Urinary tract infections (n=55)	
Yes	23 (42)
No	32 (58)
Uterine fibroids (n=62)	
Yes	25 (40)
No	37 (60)
Hysterectomy (n=62)	
Yes	22 (35)
No	40 (65)

a Response totals are noted in parentheses next to each condition. This table depicts the total number of women who reported having at least one of the health conditions listed. The most frequently reported conditions are based on the number of total responses for each health condition.

## Discussion

### Summary

With the number of women veterans on the rise, understanding their health and MEE concerns is important to address their needs. This comprehensive report captures the self-reported health problems of women with exposure histories. As part of a larger program evaluation project, a new women’s health questionnaire was developed by WOMEN CoE to serve as an addendum to the tri-WRIISC intake packet for women. The WHA was developed through an iterative process, incorporating literature review, veteran and clinician feedback, and clinical expertise. Implementation of the WHA is currently in phase 2, and results have been presented here. The respondents are women with MEE concerns seen at a tertiary VA care center, who primarily served during Gulf War I or post-9/11 eras across all branches as active-duty service members in enlisted ranks. Respondents reported several health concerns that are unique to female physiology or disproportionately impact women that were previously absent from the WRIISC intake packet. Of the nearly 97% (61/63) of women who reported at least one health condition, close to half were related to urinary health, pelvic floor dysfunction, and sexual dysfunction. Given the critical role that the pelvic floor plays in urinary and sexual health, it is not surprising that these are collectively the top health concerns. However, other concerns such as pregnancy loss and uterine fibroids were also frequently reported. Findings from this program evaluation project highlight areas of need for this patient population, ensure that women’s health issues are adequately addressed and studied, and improve health care equity for deployed veterans with MEE.

Overall, research studies examining the health of women from the Gulf War and post-9/11 eras have increased in recent years, many of which have focused on mental health [23-25]. For studies among Gulf War veterans, most have highlighted the physical and mental health status of Gulf War veterans, reported on health challenges facing Gulf War veterans such as Gulf War illness and chronic diseases, and have described the prevalence of health conditions and military exposures [17-19,25,33-36]. However, health outcomes specific to women veterans and related to reproductive health or women’s life course events have been poorly studied [18]. For studies among post-9/11 veterans, most have focused on various mental health topics (eg, posttraumatic stress disorder, military sexual trauma, and substance use disorder), assessed barriers and facilitators to health care access and use (eg, delayed care, financial concerns, and attrition), examined military exposures and combat-related stressors, and described postdeployment health (eg, chronic multisymptom illness, pain, and traumatic brain injury) [20,21,23,24,37-40]. Studies on the reproductive health of post-9/11 women veterans are also limited [22].

The results of this descriptive program evaluation project on women’s health conditions were consistent with findings from the literature. For example, Katon et al [30] also reported a high prevalence of reproductive health conditions. Although the percentage was considerably higher among WHA respondents, Katon et al [30] found that 43% of VA users had at least one reproductive health diagnosis. Moreover, among women with a diagnosis, urinary conditions (including incontinence) were the most common diagnosis across all age groups; however, sexual dysfunction and pregnancy-related conditions were also among the top diagnoses [30]. Other studies among Gulf War and post-9/11 veterans have also reported urinary conditions as top diagnoses [41,42]. In Gaffey et al [37], 26.5% of post-9/11 women veterans reported pelvic pain. Results from studies on pregnancy-related outcomes have been mixed [43]. However, Araneta et al [44] found similar adverse pregnancy outcomes among Gulf War postwar conceptions, including 22.8% spontaneous abortions, 0.7% stillbirths, and 10.7% ectopic pregnancies. In another study among Gulf War veterans [45], pregnancies ending in miscarriage/stillbirth were 25.7% for all respondents, with rates higher among Gulf War veterans compared to Gulf War–era veterans. Although VA hysterectomy rates appear to be decreasing, an elevated risk of early hysterectomy was found in one study among veterans under age 65 compared to non-veterans [22]. Among WHA respondents, 35% reported a hysterectomy. Uterine fibroids were also among the most frequently reported health conditions in this WHA sample. Even at 40%, this is likely an underestimate as many women are asymptomatic [46].

### Clinical Implications and Applications

In this clinical program evaluation, nearly 70% (36/53) of respondents stated that women’s health was important. This is not surprising given that concerns raised by women veterans were one of the many drivers of the development and implementation of the WHA. Additionally, the high percentage of women that endorsed 3 or more health conditions highlights the need for services related to women’s health, especially for this cohort of veterans with exposure histories. Furthermore, studies have shown that women with at least one reproductive health diagnosis are more likely to have concomitant medical/mental health diagnoses and have more VA outpatient encounters compared to those without a reproductive health diagnosis [22]. Although conclusions from this initial assessment are limited, future efforts to evaluate further are underway.

### Strengths and Limitations

This clinical program evaluation has several strengths. First, WOMEN CoE was able to identify a gap in the current WRIISC intake packet and take steps to address it by implementing a questionnaire to assess health outcomes that impact women veterans. WOMEN CoE clinical staff were able to incorporate veteran and clinician feedback, literature review, and clinical expertise in its development and implementation. Second, this addendum has gone through several iterations to improve the flow, length, comprehension, and coverage of important topics. At each implementation phase, changes have been identified, and modifications implemented, to improve the WHA and its response rates. Third, although these are descriptive findings and further examination is warranted, the WHA results highlight the clinical needs of women with MEE concerns seen at a WRIISC/WOMEN CoE, and the clinical team can address these by prioritizing services and resources based on these findings.

There are also limitations. One limitation is the small sample size. The sample size was not large enough to assess differences by sociodemographic, military, clinical, or other factors. With a larger sample size, we hope to conduct additional analyses to better understand our patient population. Furthermore, a larger sample size will improve future studies that aim to explore the association between MEEs reported in the WRIISC intake packet and reproductive health conditions reported in the WHA. The small sample size is driven in part by the small number of women seen at WRIISC. While the proportion of women veterans at WRIISC (18%) is higher than that of women across VA at large (9.2%) [2], as a tertiary care center, the total number of WRIISC veterans (men and women) is still small. The low response rate in phase 2 also contributes to the small sample size. At the end of phase 1, the response rate was moderate (29/75, 39%); however, the current response rate for phase 2 is low (34/325, 10%). Phase 2 is still ongoing, so the response rate might increase. Possible explanations for the low response rate include differences in survey administration, lack of familiarity with WOMEN CoE staff, and greater time since WRIISC visit. Changes to the survey modality were necessitated by the large number of women in phase 2. While WOMEN CoE staff were able to schedule appointments with former CA WRIISC patients in phase 1, by expanding the rollout to include former DC and NJ WRIISC patients, WOMEN CoE staff needed to transition to a web-based questionnaire to ensure proper scale-up. In addition, because of some overlap between CA WRIISC and WOMEN CoE clinicians, CA WRIISC patients might have been familiar with WOMEN CoE staff, which may also account for improved response rates in phase 1. Furthermore, because DC and NJ WRIISC sites were established earlier, former patients may not have had contact with WRIISC in over 23 years, which may have contributed to greater reluctance or a disinclination to respond to the invitations. Next steps include working closely with DC and NJ WRIISC staff to improve response rates among former patients and addressing barriers when expanding to new DC and NJ WRIISC patients.

Another limitation is that results from this program evaluation may not be generalizable to the typical women veteran population. At present, the WHA is specific to women with complex medical histories and exposure concerns seeking care at a specialized tertiary care center. As such, these findings may not be fully generalizable to all women seeking care at VA. Furthermore, the WHA is an addendum, and therefore, extensive information on lifetime and military exposures, military service, and other physical and mental health histories is not available without the WRIISC intake packet. However, plans to link WHA to the WRIISC intake packet are currently underway, which would allow clinicians to work closely with researchers to explore potential associations with MEEs.

Lastly, the data from this program evaluation is based on self-report, which may have introduced bias. However, several studies related to reproductive health have used patient surveys [22]. Furthermore, the WRIISC intake packet is also based on self-report, and studies have shown that self-report can be an important aspect of assessing health [47]. Given the complex medical histories of WOMEN CoE patients, it is not unreasonable to expect higher rates of self-reported health conditions compared to the general women veteran population. Additional evaluation efforts include validating self-reports with electronic medical records.

### Future Direction and Next Steps

Future modifications have already been proposed based on preliminary findings from phase 2, including changing the question format for most fill-in-the-blank questions to forced multiple choice. These results have shown that fill-in-the-blank questions have poorer response rates and often include responses that are difficult to interpret and analyze. For example, a question on the number of pregnancies will include “multiple” but not specify the number. In this example, the proposed change would include adding multiple-choice response options (eg, 1, 2, 3, 4, and 5 or more). Another proposed change includes adding additional identifier questions (eg, the last 4 digits of the social security number) to improve links to the WRIISC intake packet. Although the WHA currently includes identifier questions such as name and date of birth, changes in names due to life events (eg, marriage or divorce), typos, or variations (eg, nicknames) may pose a challenge when linking the WHA to the WRIISC intake packet, especially for former patients who completed the WRIISC intake packet many years ago.

Additional next steps include the implementation of WHA for current DC and NJ WRIISC patients. This step requires further coordination with DC and NJ centers to ensure that the questionnaire is sent within 7 days of a patient’s week-long appointment, and additional staffing to ensure timely delivery of the invitation letter and questionnaire. While these implementation barriers have delayed the phase 3 rollout, both WRIISC centers are working closely with the WOMEN CoE to address them and identify facilitators for current and future steps. Next steps also include the dissemination/expansion of the WHA to others. Several VA programs and centers beyond the WRIISC have signaled interest in using the WHA for their patient/participant populations, and some have already begun implementing the questionnaire into their project protocols on women veterans. This and future publications on the WHA are part of an effort to expand access more broadly within VA and beyond.

### Conclusion

As part of a program evaluation, WOMEN CoE clinical staff developed and implemented an addendum to the WRIISC intake packet to address the unique health concerns of women veterans. Initial findings highlight the reproductive health care needs of women with MEE concerns. As more women complete the WHA, WOMEN CoE will be able to assess the prevalence of health concerns, identify unmet health needs to prioritize, improve clinical care, evaluate potential associations of health concerns with MEEs, and disseminate findings to veterans and providers.

## Supplementary material

10.2196/73223 Checklist 1 Checklist for Reporting Results of Internet e-Surveys (CHERRIES).

## Abbreviations

CA: California
CHERRIES: Checklist for Reporting Results of Internet e-Surveys
DC: Washington, District of Columbia
MEE: military environmental exposure
NJ: New Jersey
VA: Department of Veteran Affairs
WHA: Women’s Health Addendum
WOMEN CoE: Women’s Operational Military Exposure Network Center of Excellence
WRIISC: War Related Illness and Injury Study Center
