# Supplementary material for: A Supplemental Women’s Health Questionnaire for Women Veterans With Military Environmental Exposures: Project Development and Implementation
Source: JMIR Form Res. 2025 Jul 24;9:e73223. doi: 10.2196/73223 (PMC12288700; doi:10.2196/73223)
Supplement: Checklist 1 [file formative-v9-e73223-s001.docx]

| **Item Category** | **Checklist Item** | **Explanation** | **Location** |
| --- | --- | --- | --- |
| Design | Describe survey design | Invitation-only Qualtrics survey (administered web-based and phone) | Methods section |
| IRB (Institutional Review Board) approval and informed consent process | IRB approval | Stanford IRB has reviewed and determined that this project does not meet the definition of human subject research as defined in federal regulations 45 CFR 46.102 or 21 CFR 50.3. | Methods section |
|  | Informed consent | Participants were invited via introductory letters sent to patient portal. | Methods section |
|  | Data protection | Password protected Qualtrics account for Department of Veterans Affairs (VA) was used for data collection. Data was stored behind the VA firewall. | Checklist |
| Development and pre-testing | Development and testing | Developed through an iterative process based on literature review, Veteran and clinician feedback, and clinical expertise. | Methods section |
| Recruitment process and description of the sample having access to the questionnaire | Open survey versus closed survey | Closed survey | Checklist |
|  | Contact mode | Patient portal, phone, email | Methods section |
|  | Advertising the survey | None | Checklist |
| Survey administration | Web/e-mail | Qualtrics survey administered via phone and web-based. For surveys completed via phone, data was entered manually into Qualtrics database. For surveys completed online, data was automatically transferred into the Qualtrics database. | Methods section and checklist |
|  | Context | The Women’s Health Addendum was designed using the commercial web tool Qualtrics. | Methods |
|  | Mandatory/  voluntary | Participation was voluntary | Methods section and checklist |
|  | Incentives | Incentives were not provided | Checklist |
|  | Time/date | The survey was open from October 2022 to April 2024 | Methods section |
|  | Randomization of items or questionnaires | No | Checklist |
|  | Adaptive questioning | Yes | Methods section |
|  | Number of items | 81 (maximum) | Methods section |
|  | Number of screens (pages) | 17 (minimum) | Checklist |
|  | Completeness check | We did not include completeness checks during the survey. Due to the sensitive nature of the topics covered, participants could skip questions that were triggering or elicited an unwanted reaction. | Checklist |
|  | Review step | Participants were able to review before submission | Checklist |
| Response rates | Unique site visitors | Participants who initiated the survey were considered unique site visitors (n=70). | Checklist |
|  | View rate | Not applicable | Checklist |
|  | Participation rate | In phase 1, a total of 75 participants were sent an invitation, of which 39% responded. In phase 2, 34 of the 325 (10%) invited Veterans responded. | Results section |
|  | Completion rate | The completion rate was 90% | Checklist |
| Preventing multiple entries from the same individual | Cookies used, IP check, log file analysis, and registration | Not applicable as each participant had a unique survey link, which could only be used once. | Checklist |
| Analysis | Handling of incomplete questionnaires | Participants with at least 75% of the survey completed were analyzed (n=63). Information on missing data were presented in descriptive statistics results. | Results section and checklist |
|  | Questionnaires submitted with an atypical timestamp | Not applicable | Checklist |
|  | Statistical correction | Not applicable | Checklist |
